# Corticosteroid Use and Recurrence Risk Factors in Granulomatous Mastitis: A 17-Year Saudi Arabian Cohort Study—Steroids in Granulomatous Mastitis

**DOI:** 10.3390/clinpract15100185

**Published:** 2025-10-06

**Authors:** Shoag J. Albugami, Rema F. AlRasheed, Hussam A. Alharbi, Sarah S. Alobaid, Hawazin S. Alqahtani, Mays N. Alharbi, Eyad AlKharashi, Khalid Alhajri

**Affiliations:** 1 General Surgery, Prince Sultan Military Medical City, Riyadh 12231, Saudi Arabia; ralrasheed1@psmmc.med.sa (R.F.A.); salobaid@psmmc.med.sa (S.S.A.); haalqahtani1@psmmc.med.sa (H.S.A.); mays.n.alharbi@gmail.com (M.N.A.); 2 Breast and Endocrine Surgery, Prince Sultan Military Medical City, Riyadh 12231, Saudi Arabia; aaaalharbi@psmmc.med.sa (H.A.A.); ealkharashi@psmmc.med.sa (E.A.); kalhajeri@psmmc.med.sa (K.A.)

**Keywords:** granulomatous mastitis, breast inflammatory disease, corticosteroid therapy, recurrence risk factors, Middle Eastern population

## Abstract

**Background:** Granulomatous mastitis (GM) is a rare, chronic inflammatory breast condition with poorly understood etiology and variable clinical presentation. The efficacy of corticosteroid therapy in reducing recurrence remains controversial, particularly in Middle Eastern populations where the condition appears more prevalent. This study aimed to describe the demographic and clinical characteristics of patients with GM, evaluate the efficacy of corticosteroid therapy in reducing recurrence rates, and identify risk factors associated with disease recurrence. **Methods:** A retrospective cohort analysis was conducted on 56 patients diagnosed with GM between 2003 and 2020 at a single tertiary referral center. Patients were stratified into two groups based on steroid use (n = 14 with steroids and n = 42 without steroids). **Results:** The mean age of the cohort was 46.3 ± 13.2 years, with no significant differences in baseline characteristics between the steroid and non-steroid groups. The most common presentation was a breast mass (32.69%), often associated with abscess formation (25%). Core biopsy was the primary diagnostic tool used (51.79%). Recurrence of GM occurred in 10 patients (18%) overall: 7 patients (17%) in the non-steroid group and 3 patients (21%) in the steroid group. The difference in recurrence rates between the treatment groups was not statistically significant (HR = 1.40, 95% CI:0.30–6.52, *p* = 0.671). A history of infection (HR = 5.85, 95% CI: 1.60–21.44, *p =* 0.008) and hormonal disorders (hyperprolactinemia in one patient) (HR = 13.90, 95% CI: 1.43–135.52, *p =* 0.024) were significantly associated with recurrence. **Conclusions:** GM remains diagnostically challenging with an 18% recurrence rate in our cohort. We observed no statistically significant reduction in recurrence with corticosteroids, though our analysis was limited by sample size. These findings suggest that targeted management of these conditions may be beneficial in GM patients, though larger multicenter studies are needed to confirm these associations and establish standardized treatment protocols.

## 1. Introduction

Granulomatous mastitis (GM) is a rare, chronic inflammatory condition of the breast, first described by Kessler and Wolloch in 1972 [1]. The formation of non-caseating granulomas around the breast lobules and ducts characterizes this disorder [2]. The condition is frequently associated with elevated hormonal states, such as pregnancy, breastfeeding, or oral contraceptive use, as well as autoimmune conditions [3,4,5]. Geographically, GM is more prevalent in regions such as Asia, Spain, the Middle East, and Turkey [6].

Owing to its uncertain pathogenesis and reliance on exclusionary diagnostic criteria, GM poses significant clinical challenges. It typically manifests as a unilateral painful breast mass, often accompanied by abscess formation, dermal inflammation, ulceration, nipple discharge, fistulae, and axillary lymphadenopathy. These features frequently lead to the misdiagnosis of malignant breast lesions [7]. A definitive diagnosis is achieved through histopathological examination, as imaging modalities such as ultrasound and mammography lack specific findings indicative of GM. Core needle biopsy is the preferred diagnostic tool because of its high sensitivity (94%), whereas fine-needle aspiration (FNA) is less reliable, with a sensitivity of only 21–39% [7,8,9].

Treatment strategies for GM include both surgical and non-surgical approaches, with considerable variation in practice patterns across different regions. Nonsurgical management often involves corticosteroids, immunomodulators, and antibiotics, while surgical options range from abscess drainage and wide local excision to mastectomy in severe cases [7,9]. The role of corticosteroids in GM management remains particularly controversial. While some studies have reported significant benefits in reducing inflammation and preventing recurrence [10], others have shown limited efficacy or high relapse rates after the discontinuation of steroids [11]. Yukawa et al. reported the successful management of 13 GM cases without steroid therapy [12], while Tan et al. demonstrated lower recurrence rates with oral steroid therapy [13]. These contradictory findings highlight the need for further investigation into the efficacy of corticosteroid therapy in different patient populations.

Despite numerous studies on GM, significant uncertainty remains regarding risk factors for recurrence, particularly in Middle Eastern populations. The reported recurrence rates vary widely in the literature, ranging from 5% to 50% [14,15]. Furthermore, while GM has been reported to have a higher prevalence in Middle Eastern populations [6], there is limited data on risk factors for recurrence in this region. Most existing studies have small sample sizes, heterogeneous treatment protocols, and variable follow-up periods, making it difficult to establish evidence-based management guidelines. This study specifically aimed to: (1) describe the demographic and clinical characteristics of GM patients in Saudi Arabia; (2) evaluate whether corticosteroid therapy reduces recurrence rates; and (3) identify risk factors associated with disease recurrence.

## 2. Methods

### 2.1. Study Design and Setting

This study was a retrospective cohort analysis conducted at the Breast and Endocrine Surgery Division of Prince Sultan Military Medical City in Riyadh, Saudi Arabia. The study population included consecutive patients who presented to the clinic or emergency department with a breast mass or mastitis between February 2003 and October 2020. Given the rarity of GM, this retrospective design was chosen to maximize the available sample over an extended period, allowing for long-term follow-up assessment of recurrence.

Treatment allocation was based on clinician judgment considering disease severity, patient comorbidities, and preferences. This non-randomized approach introduces potential confounding by indication, as patients receiving corticosteroids may have had more severe disease or different baseline characteristics that influenced both treatment selection and recurrence risk.

### 2.2. Patient Selection and Data Collection

Participants were identified from the department’s database and histopathology records using a systematic search for confirmed GM diagnoses. Additional detailed data were extracted from hospital electronic medical records using a standardized data collection form. All patients presenting during the study period who met the inclusion criteria and had sufficient data in their medical charts were included, resulting in a final sample size of 56 patients. The inclusion criteria were: (1) histopathologically confirmed diagnosis of GM; (2) age ≥18 years; and (3) minimum follow-up of 6 months after initial treatment. We excluded patients with: (1) missing essential data; (2) non-confirmed diagnosis of GM; (3) associated breast cancer; and (4) loss to follow-up before 6 months. The patients were divided into two groups based on whether they received steroid therapy (n = 14) or not (n = 42). Treatment allocation was based on clinician judgment and patient factors, including disease severity, comorbidities, and patient preferences, reflecting real-world clinical practice.

### 2.3. Study Variables and Definitions

Data collection included demographic details such as age at diagnosis, sex, body mass index (BMI), marital status, age at menarche, number of pregnancies, breastfeeding history, and oral contraceptive pill (OCP) use. Clinical features, radiological and histopathological findings, treatment modalities, and recurrence rates were recorded. Follow-up data were collected from the electronic patients’ charts, including clinic visits, imaging studies, and any subsequent interventions.

The primary outcome measured was GM recurrence, defined as the reappearance of clinical symptoms and signs of GM (breast mass, pain, inflammation, or abscess) after a symptom-free period of at least 6 months, confirmed by clinical examination and, when available, imaging or histopathology. Secondary outcomes included treatment-related complications and the development of malignancy during follow-up.

Key variables were defined as follows: History of infection: Documented evidence of previous breast infection or abscess before GM diagnosis. Hormonal disorders: Diagnosed conditions affecting hormonal balance (e.g., hyperprolactinemia, thyroid dysfunction, polycystic ovary syndrome). Autoimmune disease: Diagnosed autoimmune conditions (e.g., rheumatoid arthritis, systemic lupus erythematosus).

### 2.4. Treatment Protocol

Patients in the steroid group received oral prednisolone at an initial dose of 0.8 mg/kg/day for 2–4 weeks, followed by a gradual taper over 2–3 months based on clinical response. The typical tapering schedule involved dose reduction by 5–10 mg every 1–2 weeks, with slower tapering for patients showing signs of symptom recurrence during dose reduction. Patients with diabetes received modified dosing schedules with closer monitoring of blood glucose levels. Most patients received empirical antibiotic therapy, typically amoxicillin-clavulanate or clindamycin for patients with penicillin allergy, for a minimum of 7–14 days. Surgical interventions, including incision and drainage of abscesses or excision of persistent masses, were performed as clinically indicated. Follow-up visits were scheduled at 2 weeks, 1 month, 3 months, and then every 6 months for at least 2 years.

### 2.5. Ethical Considerations

The study was conducted in accordance with the Declaration of Helsinki and approved by the Institutional Review Board at Prince Sultan Military Medical City. The need for patient consent was waived because of the retrospective design and the use of de-identified data. Patient confidentiality was maintained throughout the data collection and analysis process.

### 2.6. Statistical Analysis

For Statistical Analysis: Stata 18 (Stata Corp, College Station, TX, USA) was used for statistical analysis. Continuous data are expressed as mean ± standard deviation or median (25th–75th percentiles), depending on the normality of the distribution. Comparisons between groups were made using Student’s *t*-test or the Mann–Whitney test, as appropriate. Categorical data are presented as numbers and frequencies, and comparisons were made using the chi-squared or Fisher’s exact test. Time-to-event data were analyzed using Kaplan–Meier curves, and the log-rank test was used for comparisons. Univariate Cox regression analysis was performed to identify factors associated with recurrence. Missing data were considered missing at random, and complete case analysis was performed. Statistical significance was set at *p* < 0.05.

The study was reported according to the STROBE (Strengthening the Reporting of Observational Studies in Epidemiology) guidelines for cohort studies [16].

## 3. Results

### 3.1. Baseline Data

Table 1 summarizes the baseline demographic and clinical characteristics of the study population. The mean age of the study cohort was 46.3 ± 13.2 years, and the median BMI was 28 (26–30) kg/m^2^. The majority of patients were married (94.64%) and had a history of breastfeeding (82.14%). There were no statistically significant differences in age, BMI, smoking status, marital status, menstrual history, or obstetric history between the steroid and non-steroid groups. Seven patients (12.50%) had a history of infection, with a higher proportion in the steroid group (21.43%) compared to the non-steroid group (9.52%), though this difference was not statistically significant (*p* = 0.350).

### 3.2. Presentation and Management

The clinical presentation and management strategies are summarized in Table 2. The most common clinical presentation was a breast mass, which was observed in 17 patients (32.69%). A breast mass with an associated abscess was the second most common presentation, occurring in 13 patients (25%). There was no statistically significant difference in the pattern of presentation between patients with and without steroid therapy (*p* = 0.295).

Regarding anatomical location, the upper outer quadrant (UOQ) was the most commonly affected site (27.27%), followed by the upper inner quadrant (UIQ, 16.36%) and lower inner quadrant (LIQ, 12.73%). Core biopsy was the most common diagnostic method used (51.79%), followed by fine-needle aspiration (FNA, 21.43%) and tissue biopsy (14.29%). Some patients underwent multiple biopsy types. The most common imaging finding was a mass (35.19%), followed by collections/abscesses (7.41%) and asymmetry (7.41%). For 14 patients (25.93%), imaging studies were not performed or available for review at our institution, as diagnosis was confirmed through histopathological analysis of biopsy specimens. No significant differences in biopsy type or imaging findings were observed between the treatment groups.

Incision and drainage procedures were performed in 35 patients (63.64%), with similar rates in both treatment groups (*p* = 0.839). Nearly all patients (98.21%) received antibiotic therapy.

### 3.3. Follow-Up and Recurrence

**Figure 1:**
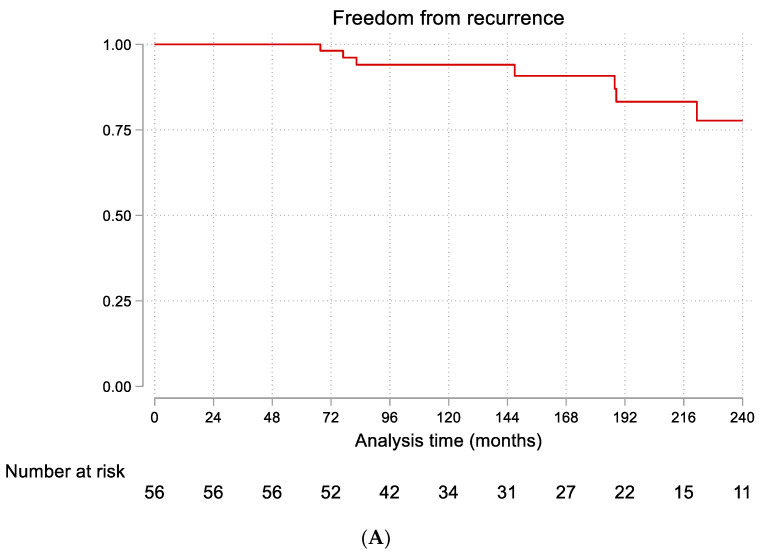

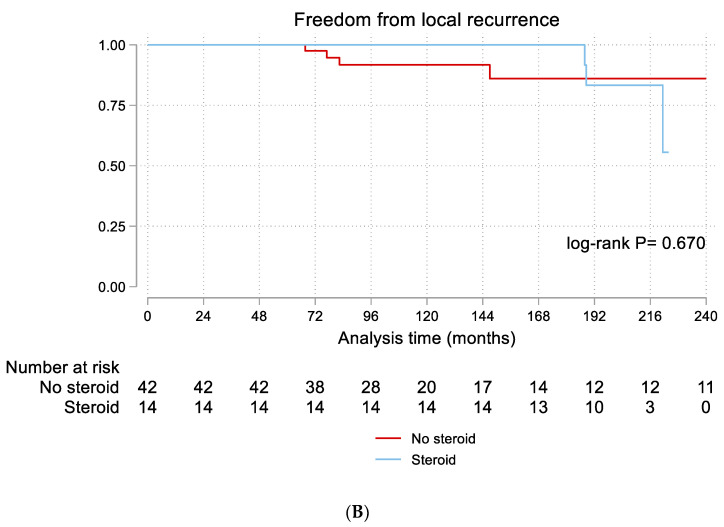
Kaplan–Meier curves for freedom from local recurrence. (**A**) All patients (n = 56). (**B**) Comparison between steroid-treated (n = 14, blue line) and non-steroid-treated patients (n = 42, red line).

The median follow-up period was 155 months (25th–75th percentiles: 100–223 months). During follow-up, malignancy was diagnosed in three patients (5.36%), all of whom were in the non-steroid group. These malignancies were diagnosed at 24, 36, and 48 months after the initial GM diagnosis, respectively. Recurrence of GM occurred in 10 patients (18%) overall, including the patient with a hormonal disorder: 7 patients (17%) in the non-steroid group and 3 patients (21%) in the steroid group. The difference in recurrence rates between the treatment groups was not statistically significant (HR = 1.40, 95% CI: 0.30–6.52, *p* = 0.671). Figure 1A illustrates the Kaplan–Meier curve for freedom from local recurrence in the entire cohort, showing rates of 98% at 5 years, 94% at 10 years, and 78% at 20 years. Figure 1B compares recurrence-free survival between the steroid and non-steroid groups, demonstrating no significant difference (log-rank *p* = 0.670).

### 3.4. Risk Factors for Recurrence

Univariate Cox regression analysis was performed to identify factors associated with GM recurrence (Table 3). A history of infection emerged as a significant risk factor for recurrence (HR = 5.85, 95% CI: 1.60–21.44, *p* = 0.008). Similarly, hormonal disorders were strongly associated with recurrence (HR = 13.90, 95% CI: 1.43–135.52, *p* = 0.024). However, this finding is based on a single patient with hyperprolactinemia who experienced a recurrence and should therefore be interpreted with extreme caution due to the statistical instability of the estimate.

Other factors showing trends toward association with recurrence, though not reaching statistical significance, included higher BMI (HR = 1.17, 95% CI: 0.98–1.27, *p* = 0.091), younger age at menarche (HR = 0.65, 95% CI: 0.39–1.05, *p* = 0.078), absence of breastfeeding history (HR = 0.33, 95% CI: 0.08–1.23, *p* = 0.098), presence of autoimmune disease (HR = 2.99, 95% CI: 0.80–11.22, *p* = 0.104), and absence of incision and drainage procedures (HR = 0.16, 95% CI: 0.02–1.27, *p* = 0.084). Notably, steroid therapy did not significantly affect recurrence risk (HR = 1.40, 95% CI: 0.30–6.52, *p* = 0.671).

## 4. Discussion

Granulomatous mastitis (GM) remains a challenging inflammatory breast condition with significant diagnostic and therapeutic complexities. This retrospective cohort study provides insights into the clinical characteristics, treatment outcomes, and recurrence risk factors of GM in a Saudi Arabian population over a 17-year period. Our findings contribute to the growing body of evidence on GM management, particularly in Middle Eastern populations where the condition appears to be more prevalent but remains understudied.

### 4.1. Clinical Characteristics and Presentation

The mean age of our cohort was 46.3 years. This is somewhat higher than the mean age typically reported in the literature for patients with granulomatous mastitis. Al-Khaffaf et al. reported a mean age of 36 years in their series of 133 patients [15]. This variation in age distribution across different populations highlights the heterogeneity of GM and suggests potential regional differences in disease etiology or risk factors. The predominance of GM in women of reproductive age across studies supports the hypothesis that hormonal factors play a role in its pathogenesis [17].

The clinical presentation in our cohort was consistent with previous reports, with breast mass being the most common manifestation, often accompanied by abscess formation. This presentation pattern can mimic inflammatory breast cancer, underscoring the diagnostic challenges associated with GM [18,19,20]. The predilection for the upper outer quadrant observed in our study aligns with findings from other series [21] and may reflect the greater volume of glandular tissue in this region.

While no statistically significant differences were observed in measured baseline characteristics between treatment groups, unmeasured confounders related to disease severity or clinical presentation may have influenced treatment allocation.

### 4.2. Diagnostic Approach

Our findings confirm the central role of histopathological examination in establishing a definitive diagnosis of GM. Core biopsy was the most frequently employed diagnostic method in our cohort (51.79%), consistent with recommendations in the literature due to its high sensitivity (94%) [9]. The limitations of imaging modalities in providing specific findings for GM were evident in our study, with masses and collections being the predominant but nonspecific findings. This reinforces the importance of tissue diagnosis to differentiate GM from malignancy and other inflammatory conditions [20].

### 4.3. Treatment Efficacy and Recurrence

A key finding of our study was the lack of significant difference in recurrence rates between patients treated with and without corticosteroids. This contrasts with some previous reports suggesting beneficial effects of steroid therapy. Tan et al. demonstrated lower recurrence rates with oral steroid therapy in their cohort [13]. However, our findings align with other studies showing limited efficacy of corticosteroids in preventing recurrence [22]. Several factors might explain this discrepancy, including the nonrandom treatment allocation in our study. Our steroid protocol differs from some published regimens that use higher initial doses [23], and our sample size is small, particularly in the steroid group.

The overall recurrence rate of 18% in our cohort falls within the range reported in the literature (5–50%) [14,15]. The long-term follow-up in our study (median 155 months) provides valuable data on the natural history of GM, showing that while most recurrences occur within the first few years, late recurrences are possible, with freedom from recurrence decreasing from 98% at 5 years to 78% at 20 years. While our study identified key risk factors for overall recurrence, a further area of investigation is the timing of recurrence (i.e., early vs. late). A key question is whether risk factors such as a history of infection or hormonal disorders are associated with an earlier onset of recurrence. Unfortunately, due to the limited number of recurrence events in our study, we were unable to perform a formal statistical analysis to address this important question. Such an analysis would be underpowered and at risk of producing spurious findings. However, this is a critical area for future research.

An unexpected finding in our study was that all three cases of malignancy diagnosed during follow-up occurred in the non-steroid group. We believe this observation could be due to chance, given the small number of events.

It is worth mentioning that the frequent use of antibiotics (98.21%) and incision and drainage procedures (63.64%) as co-interventions makes it impossible to isolate the independent effect of corticosteroids. These interventions were distributed similarly between groups but represent important confounding factors in assessing corticosteroid efficacy

### 4.4. Risk Factors for Recurrence

Perhaps the most clinically relevant finding of our study was the identification of specific risk factors for GM recurrence. A history of infection emerged as a significant predictor. This association supports the infectious etiology hypothesis for GM [24] and suggests that thorough microbiological evaluation and targeted antimicrobial therapy might be beneficial in selected patients.

Similarly, while we found a statistically significant association between hormonal disorders and recurrence, this was based on only one patient with hyperprolactinemia. The resulting hazard ratio is therefore highly unstable, as reflected by the extremely wide confidence interval, and we caution against over-interpreting this finding. Although the link between hormonal factors and GM is well-established, our data do not allow for any firm conclusions on this specific risk factor. Future studies with larger sample sizes are needed to investigate the role of specific hormonal conditions, such as thyroid disease or polycystic ovary syndrome, on the risk of GM recurrence. This finding aligns with the established link between hormonal factors and GM pathogenesis [25] and suggests that hormonal evaluation and potential intervention might be considered in GM management.

### 4.5. Potential Mechanisms Underlying Recurrence Risk Factors

Hormonal Pathways in GM Recurrence:

Our finding that hormonal disorders (based on one patient) and OCP use are associated with GM recurrence aligns with a growing body of evidence implicating hormonal imbalances in the pathogenesis of this disease. One of the most frequently cited hormonal factors is hyperprolactinemia. Prolactin is a key hormone in mammary gland development and lactation, and its receptors are highly expressed in breast tissue. It is hypothesized that elevated prolactin levels may promote ductal ectasia and stasis of secretions, creating a pro-inflammatory environment that can trigger a granulomatous reaction. Furthermore, prolactin has immunomodulatory effects, and its dysregulation could contribute to the aberrant immune response seen in GM [13,15,20]. While we did not systematically measure prolactin levels in our cohort, the association with hormonal disorders suggests that this pathway warrants further investigation. Future studies should include comprehensive hormonal profiling to elucidate the specific roles of prolactin, estrogen, and progesterone in GM pathogenesis and recurrence.

Infection, Inflammation, and Microbial Factors:

The strong association between a history of infection and GM recurrence in our study suggests a potential role for microbial triggers and chronic inflammation. It is possible that an initial infection, even if clinically resolved, may lead to a persistent, low-grade inflammatory state or an altered local microbiome. This could create a state of immune dysregulation where a subsequent trigger can elicit an exaggerated granulomatous response. Some researchers have proposed that specific microorganisms, such as *Corynebacterium* species, may be involved in the pathogenesis of GM, potentially by forming biofilms that are resistant to conventional antibiotic therapy [24]. While our study was not designed to investigate microbial etiologies, our findings underscore the importance of considering the interplay between infection and inflammation.

Autoimmune Mechanism

In our study, a history of autoimmune disease showed a trend toward an increased risk of recurrence (HR = 2.99, *p* = 0.104), although this did not reach statistical significance. This finding, while not conclusive, aligns with the growing hypothesis that GM may be an autoimmune or autoinflammatory condition in a subset of patients [4]. The underlying immunological mechanisms could involve a dysregulation of both innate and adaptive immunity.

It is plausible that in patients with a pre-existing autoimmune diathesis, the immune system is already primed for an exaggerated response to certain triggers, such as hormonal fluctuations or microbial antigens. This could lead to an overactivation of T-helper 1 (Th1) cells, which are known to drive granuloma formation through the secretion of pro-inflammatory cytokines like interferon-gamma (IFN-γ) and tumor necrosis factor-alpha (TNF-α). A breakdown in peripheral tolerance mechanisms, which is a hallmark of autoimmune diseases, could also allow for the development of an autoimmune response directed against self-antigens in the breast tissue [19,20].

### 4.6. International Context and Regional Differences in GM

Our study provides insights into GM’s clinical characteristics and recurrence patterns in a Saudi Arabian cohort. When comparing our findings to those from other regions, several interesting differences emerge. For example, the mean age of our cohort (46.3 years) is higher than that reported in some Turkish and Iranian studies, where the mean age is often in the mid-30s [26,27]. This could reflect differences in reproductive patterns, environmental exposures, or genetic predisposition.

Our overall recurrence rate of 18% is within the wide range reported in the literature (5–50%). However, some studies from Singapore have reported lower recurrence rates, which may be attributable to different treatment protocols that could include more aggressive use of corticosteroids or immunomodulators [13]. Conversely, some reports from Iran have shown higher recurrence rates [23,28].

Several factors may contribute to these regional differences. Genetic factors, such as specific HLA haplotypes, may predispose certain populations to GM or to a more aggressive disease course. Environmental factors, including diet, lifestyle, and exposure to specific microbial agents, may also play a role. Cultural and reproductive factors, such as breastfeeding practices and the use of hormonal contraceptives, can vary significantly between regions and may influence the incidence and recurrence of GM. Finally, differences in healthcare systems and clinical practice patterns can also impact outcomes. The diagnostic workup, treatment algorithms, and intensity of follow-up can all vary, making direct comparisons between studies challenging.

### 4.7. Limitations

This study has several important limitations. First, the retrospective design introduces potential selection and information biases. Second, the small sample size (n = 56) and limited number of recurrence events (n = 10) restricted our analysis to univariate methods, resulting in wide confidence intervals around the effect estimates. Third, we acknowledge the potential for confounding by indication in our study, as treatment was not randomized. Patients in the steroid group had a higher (though not statistically significant) prevalence of infection history and OCP use, suggesting they may have had more severe or hormonally influenced disease. Another significant confounder is the treatment protocol heterogeneity. The near-universal use of antibiotics and the high frequency of incision and drainage procedures are significant co-interventions that obscure the ability to determine the isolated effect of corticosteroid therapy. Fourth, data were collected from a single center in Saudi Arabia, which may limit generalizability to other populations. Fifth, the long study period (2003–2020) may have introduced historical bias due to changes in diagnostic and treatment practices over time. Finally, a major limitation of our study is the small sample size, particularly in the corticosteroid treatment group (n = 14). This resulted in the study being significantly underpowered to detect a clinically meaningful difference in recurrence rates between the steroid and non-steroid groups. Therefore, while we did not find a statistically significant effect of steroid therapy, this finding should be interpreted with caution, and we cannot rule out a true clinical benefit (or harm) of corticosteroids. Larger, multicenter, randomized controlled trials are urgently needed to definitively determine the efficacy of corticosteroids in the management of granulomatous mastitis and to provide robust evidence to guide clinical practice. These limitations necessitate cautious interpretation of our findings. The associations identified should be considered hypothesis-generating rather than definitive, and the lack of difference between treatment groups should not be interpreted as evidence of no effect, as our study was underpowered to detect modest treatment benefits. The retrospective nature of our study resulted in missing data for several variables. Complete case analysis was performed, which assumes data are missing completely at random. Patients with missing data may represent a different population subset, potentially affecting the generalizability of our findings.

## 5. Conclusions

GM is a complex and diagnostically challenging condition that often mimics malignant breast lesions and leads to significant clinical and therapeutic dilemmas. This study provides valuable insights into the demographic and clinical characteristics of patients with GM in a Saudi Arabian cohort, highlighting the high recurrence rate and the limited impact of corticosteroid therapy on reducing recurrence. The identification of infections and hormonal disorders as significant risk factors for recurrence underscores the need for a more nuanced understanding of disease pathogenesis and the development of targeted treatment strategies. Although corticosteroids remain a common therapeutic option, their efficacy in preventing recurrence is limited, suggesting that alternative or adjunctive therapies should be explored. Given the methodological limitations, particularly confounding by indication and co-interventions, our findings cannot establish causal relationships between corticosteroid use and recurrence outcomes. Future randomized controlled trials are needed to definitively assess corticosteroid efficacy in granulomatous mastitis.

## Figures and Tables

**Table 1:** Comparison of baseline data between patients with granulomatous mastitis with and without steroids.

Variables	Total (n = 56)	No Steroid (n = 42)	Steroid (n = 14)	*p*-Value
Age (years)	46.3 ± 13.2	46.3 ± 13.9	48.1 ± 11	0.648
Body mass index (kg/m^2^)	28 (26–30)	29 (27–31)	27 (26–28)	0.061
Smokers	1 (1.79%)	1 (2.38%)	0	>0.99
Married	53 (94.64%)	39 (91.86%)	14 (100%)	0.565
Age of menarche (n = 52) (years)	12.7 ± 1.7	12.9 ± 1.6	12.3 ± 1.6	0.349
Menopause	26 (46.43%)	20 (47.62%)	6 (42.86%)	0.757
History of oral contraceptive pills	28 (50%)	18 (42.86%)	10 (71.43%)	0.064
Number of pregnancies	5 (3–7)	5 (3–8)	5 (3–6)	0.972
History of breast feeding	46 (82.14%)	36 (85.71%)	10 (71.43%)	0.247
Family history of breast cancer	11 (19.64%)	7 (16.67%)	4 (28.57%)	0.332
History of infections	7 (12.50%)	4 (9.52%)	3 (21.43%)	0.350
Autoimmune disease	13 (23.21%)	9 (21,43%)	4 (28.57%)	0.584
Antidepressant	3 (5.36%)	2 (4.76%)	1 (7.14%)	>0.99
Hormonal disorders (Hyperprolactinemia)	1 (1.79%)	1 (2.38%)	0	>0.99
Extrinsic trauma	4 (7.14%)	4 (9.52%)	0	0.562

Data are presented as mean (SD) or number (%).

**Table 2:** Comparison of presentation and diagnosis data between patients with granulomatous mastitis with and without steroids.

Variables	Total (n = 56)	No Steroid (n = 42)	Steroid (n = 14)	*p*-Value
Presentation (n = 52)				0.295
Mass	17 (32.69%)	14 (36.84%)	3 (21.43%)	
Mass + abscess	13 (25%)	8 (21.05%)	5 (35.71%)	
Mass + abscess + pain + nipple changes	1 (1.92%)	0	1 (7.14%)	
Mass + abscess + ulcer	1 (1.92%)	1 (2.63%)	0	
Mass + pain	4 (7.69%)	4 (10.53%)	0	
Mass + pain + fistula	4 (7.69%)	2 (5.26%)	2 (14.29%)	
Mass + nipple discharge or change	1 (1.92%)	1 (2.63%)	0	
Mass + fistula	1 (1.92%)	0	1 (7.14%)	
Abscess	2 (3.85%)	2 (5.26%)	0	
Pain	8 (15.38%)	6 (15.79%)	2 (14.29%)	
Location				0.18
Not documented	13 (23.21%)	12 (28.57%)	1 (7.14%)	
UOQ	15 (26.79%)	11 (26.83%)	4 (28.57%)	
LOQ	6 (10.71%)	3 (7.32%)	3 (21.43%)	
LOQ + LIQ	1 (1.79%)	1 (2.44%)	0	
UIQ	9 (16.07%)	7 (17.07%)	2 (14.29%)	
UIQ + LIQ	1 (1.79%)	1 (2.44%)	0	
LIQ	7 (12.50%)	3 (7.32%)	4 (28.57%)	
Retroareolar	4 (7.14%)	4 (9.76%)	0	
Side				0.162
Right	25 (44.64%)	21 (50%)	4 (28.57%)	
Left	31 (55.36%)	21 (50%)	10 (71.43%)	
Biopsy type				0.441
FNA	12 (21.43%)	10 (23.81%)	2 (14.29%)	
FNA + core biopsy	4 (7.14%)	4 (9.52%)	0	
FNA + tissue biopsy	2 (3.57%)	2 (4.76%)	0	
FNA + core + tissue biopsy	1 (1.79%)	1 (2.38%)	0	
Core biopsy	29 (51.79%)	18 (42.86%)	11 (78.57%)	
Tissue biopsy	8 (14.29%)	7 (16.67%	1 (7.14%)	
Imaging findings				0.483
Asymmetry	4 (7.41%)	4 (10%)	0	
Asymmetry + mass	1 (1.85%)	0	1 (7.14%)	
Mass	19 (35.19%)	14 (35%)	5 (35.71%)	
Mass + collections/abscess	1 (1.85%	1 (2.5%)	0	
Collections/abscess	4 (7.41%)	4 (10%)	0	
Others	11 (20.37%)	7 (17.5%)	4 (28.57%)	
No images	14 (25.93%)	10 (25%)	4 (28.57%)	
Incision and drainage	35 (63.64%)	26 (63.41%)	9 (64.29%)	0.839
Antibiotic use	55 (98.21%)	41 (9762%)	14 (100%)	>0.99

FNA, fine needle aspiration; LIQ, lower inner quarter; LOQ, lower outer quadrant; UIQ, upper inner quadrant; UOQ, upper outer quadrant. Data were presented as number (%).

**Table 3:** Univariable Cox regression for factors associated with local recurrence.

Variables	HR (95% CI)	*p*
Demographics		
Age	0.98 (0.92–1.04)	0.577
Body mass index	1.17 (0.98–1.27)	0.091
Married	0.59 (0.07–4.85)	0.622
Age of menarche	0.65 (0.39–1.05)	0.078
Menopause	0.28 (0.06–1.29)	0.103
Clinical factors		
History of oral contraceptive pills	1.13 (0.32–4.06)	0.849
Number of pregnancies	0.95 (0.76–1.21)	0.709
History of breast feeding	0.33 (0.08–1.23)	0.098
Family history of breast cancer	2.77 (0.34–22.26)	0.338
History of infections	5.85 (1.60–21.44)	0.008
Autoimmune disease	2.99 (0.80–11.22)	0.104
Hormonal disorders (Hyperprolactinemia)	13.90 (1.43–135.52)	0.024
Anatomical factors		
Location	1.44 (0.88–2.34)	0.139
Side	1.27 (0.36–4.53)	0.710
Imaging findings	1.29 (0.84–1.98)	0.243
Treatment		
Incision and drainage	0.16 (0.02–1.27)	0.084
Steroid	1.40 (0.30–6.52)	0.671

HR: hazard ratio, CI: confidence interval.

## Data Availability

Data sharing requires institutional approval according to the institutional regulations. The data presented in this study are available upon request from the corresponding author. The data are not publicly available due to institutional privacy and ethical restrictions.
